# Food allergy associated to Parkinson diseases in Venezuelan patients

**DOI:** 10.1186/2045-7022-5-S3-P140

**Published:** 2015-03-30

**Authors:** Franca Puccio, R Rojas, I Mosquera, D Cifarrelli, A Hernández, M Lizarralde, G Peña, L Jaua, C Mendoza, R Reyes

**Affiliations:** 1 Instituto de Biomedicina-MSDS, UCV, Caracas, Venezuela; 2 Escuela de Nutrición y Dietética, Caracas, Venezuela; 3 Clínica de Dolor Crónico y Fibromialgia, Instituto de Neurología y Neurociencias Aplicadas, Caracas, Venezuela

## Background

The association with Parkinson’s disease is increased with allergic rhinitis. Increasing evidence supports an important role of central and peripheral inflammation in driving Parkinson’s (PD) initiation and progression. The link between peripheral inflammation and neurodegeneration in PD patients has been revealed in several clinical reports. In addition, gut inflammation mediated by Th2 responses occurs in PD patients. However, the evaluation and study of allergic symptoms was not commonly performed in patients with Parkinson (PD).

## Objective

The aim of this study was to evaluated Allergy symptoms, the main food allergens and specific IgE to food allergens in Parkinson patients.

## Methods

We evaluated 92 Parkinson patients who assisted to Neurological and Neurosciences Institute, Caracas, Venezuela, by a multidisciplinary team work; we performed physical examination including neurological and cognitive evaluation. Patient were classified according to the criteria of the International Consensus Report as suffering rhinitis only, asthma without significant rhinitis, and combined asthma and rhinitis as defined by the guidelines of the WHO/NIH Global Initiative for Asthma (GINA). Patients with atopic dermatitis were classified following Hanifin and Rajka. Food allergy induces symptoms were evaluated in a medical history and physical examination according to NIAD (Guidelines for the Diagnosis and Managementof Food Allergy). Skin prick tests (SPT) were performed with a battery of commercial allergens (ALK-Abello) extracts according to international guidelines. Specific IgE to common food allergens were detected. Sleep quality were evaluate using a patient interview and sensitivity analyses using a scored visual and analogue scale, and polysomnography study were performed.

## Results

We found that 45% of patients had rhinitis, 8.77% asthma and food allergy symptoms were present in 49% of cases.46% of patient reports different symptoms with milk, orange and wheat. Specific milk proteins IgE were positive in a 40% of patients with gastrointestinal symptoms. Ninety percent of PD patients had sleep disorders. Allergy condition and food allergy to common food, were important features to evaluate as associated diseases to Parkinson’s in order to improve better treatment to the patients.

